# Preliminary Study of In Vitro Three-Dimensional Skin Model Using an Ovine Collagen Type I Sponge Seeded with Co-Culture Skin Cells: Submerged versus Air-Liquid Interface Conditions

**DOI:** 10.3390/polym12122784

**Published:** 2020-11-25

**Authors:** Mh Busra Fauzi, Zahra Rashidbenam, Aminuddin Bin Saim, Ruszymah Binti Hj Idrus

**Affiliations:** 1 Centre for Tissue Engineering and Regenerative Medicine, Faculty of Medicine, UKM Medical Centre, Jalan Yaacob Latiff, Bandar Tun Razak, Kuala Lumpur 56000, Malaysia; zahra.rashidbenam@gmail.com (Z.R.); ruszyidrus@gmail.com (R.B.H.I.); 2 Ear, Nose & Throat Consultant Clinic, Ampang Puteri Specialist Hospital, Taman Dato Ahmad Razali, Selangor 68000, Malaysia; aminuddinsaim@gmail.com; 3 Department of Physiology, Faculty of Medicine, UKM Medical Centre, Jalan Yaacob Latiff, Bandar Tun Razak, Kuala Lumpur 56000, Malaysia

**Keywords:** ovine collagen, collagen type I, genipin, carbodiimide, 3D skin model, air-liquid interface

## Abstract

Three-dimensional (3D) in vitro skin models have been widely used for cosmeceutical and pharmaceutical applications aiming to reduce animal use in experiment. This study investigate capability of ovine tendon collagen type I (OTC-I) sponge suitable platform for a 3D in vitro skin model using co-cultured skin cells (CC) containing human epidermal keratinocytes (HEK) and human dermal fibroblasts (HDF) under submerged (SM) and air-liquid interface (ALI) conditions. Briefly, the extracted OTC-I was freeze-dried and crosslinked with genipin (OTC-I_GNP) and carbodiimide (OTC-I_EDC). The gross appearance, physico-chemical characteristics, biocompatibility and growth profile of seeded skin cells were assessed. The light brown and white appearance for the OTC-I_GNP scaffold and other groups were observed, respectively. The OTC-I_GNP scaffold demonstrated the highest swelling ratio (~1885%) and water uptake (94.96 ± 0.14%). The Fourier transformation infrared demonstrated amide A, B and I, II and III which represent collagen type I. The microstructure of all fabricated sponges presented a similar surface roughness with the presence of visible collagen fibers and a heterogenous porous structure. The OTC-I_EDC scaffold was more toxic and showed the lowest cell attachment and proliferation as compared to other groups. The micrographic evaluation revealed that CC potentially formed the epidermal- and dermal-like layers in both SM and ALI that prominently observed with OTC-I_GNP compared to others. In conclusion, these results suggest that OTC_GNP could be used as a 3D in vitro skin model under ALI microenvironment.

## 1. Introduction

Recently, the use of non-animal models to evaluate the safety of materials in industry, agriculture and biomedical applications has gradually increased [1]. The acute dermal toxicity, skin irritation/corrosion, skin sensitisation, acute oral toxicity, acute inhalation toxicity and eye irritation/corrosion have been recognised as basic six safety testing packages [2]. The first three testing involve skin-related tissue as a major element in the safety evaluation. Human skin is a complex external barrier in terms of its physiology and microstructure. However, developing a mature reconstructed human skin (RHS) construct that can function long-term for conducting tests in vitro has been a limitation, even with the combination of two main types of skin cells [3]. Current commercially available in vitro skin models are only valid for one week to run the safety test. To date, there has been a lack of information on developing three-dimensional (3D) in vitro skin models using direct seeding of co-cultured skin cells on a collagen scaffold, especially ovine collagen type I.

Briefly, the current RHS has been used for various applications in the cosmeceutical, theranostic, pharmaceutical, biomedical and clinical settings. Moreover, RHS has been useful as an in vitro model for toxicity testing drugs, chemical and advanced materials. The Organisation for Economic Cooperation and Development (OECD) guideline 428 clearly mentions that the preparation of human skin, including full-thickness skin, split-thickness skin and heat-separated human epidermis, can be imitated by an in vitro human skin equivalent. However, the quality of reconstructed 3D skin should be comparable with human skin, so any further testing would be closely related to native skin.

Traditionally, safety testing is performed in animal models, such as rodents (rats and mice) or non-rodents (dogs and mini pigs) because they represented systemic organisms resembling physiologically and functionally as of human being [3]. However, these models have several limitations to be considered, including high cost, limited interventions per animal, ethical considerations in certain countries and species variation that ultimately need supporting data from the abovementioned animal models to be sufficient [4]. For over a century, two-dimensional (2D) cell cultures have been used as in vitro models to study cellular responses to stimuli from biophysical and biochemical cues [5]. Thus far, plenty of materials had been studied for their application in skin tissue engineering. For instance, Zein, which is a plant-based protein, in combination with polycaprolactone and gum Arabic, has been recently studied and the outcomes showed favourable hydrophilicity, porosity, tensile strength and elongation of the composite material for skin tissue engineering [6]. However 2D skin cell cultures come with an overt lack of physiological relevance. Most principal functions of skin, like barrier function, resilience, cell sheeting, cell layering, development profiles, immune function, blood perfusion and innervation are not recapitulated in simple 2D cultures [7].

The innovative medical and material research has led to novel technologies in the quest for improved cell culture methods. Pioneering research in three-dimensional (3D) culture models from the Bissell laboratory has opened the way for more sophisticated and relevant culture models than the traditional 2D methods [8]. 3D cell cultures differ greatly from standard 2D cell cultures in cell-cell interactions, cellular mechanics and nutrient access [9]. Briefly, 3D skin equivalents are organotypic culture systems that are generated by seeding human keratinocytes onto a dermal layer with human fibroblasts [10]. The positive trend of increasing acceptance of these models in dermatological research will hopefully lead to a reduction in the number of animals used for experiments to study skin biology, wound healing, skin ageing and disease pathology [11,12]. In addition, 3D skin equivalents have been developed as an attractive tool for pharmacotoxicological testing of cosmetic components, for clinical applications such as artificial skin grafts and to avoid the need for sequential invasive skin biopsies from patients for standardised studies of human skin [10,13].

Currently, 3D skin models can be generated by culturing keratinocytes onto a cellular matrix (fibroblast-collagen matrix), acellular matrix (de-epidermised dermis, DED) or inert plastic filler. As a replacement to chemically synthesized biopolymers, such as chitosan [14] and sodium alginate [15], had also been investigated for their potential application in 3D skin tissue engineering. Tchemtchoua and co-workers [16] have reported successful results for in vitro and in vivo application of chitosan when it is used as nanofibre scaffold (2D). However, chitosan, when it is used in sponge format (3D), causes foreign body reaction and was not favouring vascularisation. The selection of the bioscaffold plays a crucial role in 3D skin model development to provide an appropriate base for tissue growth and proliferation [17]. The 3D scaffold has to be biocompatible, non-toxicity, non-immunogenic and biodegradable, and in order to have a successful engraftment when it is applied in clinical setting the 3D scaffold should be able to support vascularization [18]. Additionally, 3D scaffolds should provide sufficient mechanical strength (similar to native tissue) and a crosslinked network structure [19] that is sustained throughout the incubation period. This has been widely applied clinically in various forms such as gels or sponges due to their biodegradability and absorbability [20]. Niehues and co-workers have reported the successful formation of a multilayered stratified epithelium, resembling skin in vivo, which was grown under submerged and air-liquid interface (ALI) conditions for two weeks [12].

Previous studies have described the successful extraction of collagen type I from ovine tendon. Ovine collagen type I is able to form various 3D scaffold designs, and its application as a rapid treatment for full thickness skin loss has been explored [21,22,23]. Thus, this study aimed to develop an RHS using a lyophilised ovine tendon collagen type I (OTC-I) bioscaffold with different crosslinking agents, i.e., natural (genipin (GNP)) and synthetic (carbodiimide (EDC)), which was followed by co-culture of human skin cells under submerged (SM) and air liquid interface (ALI) conditions. Moreover, the physico-chemical properties of the OTC-I bioscaffolds were characterised. In addition, we evaluated the cellular biocompatibility of OTC-I bioscaffolds and their ability in supporting the formation of epidermal- and dermal-like skin structures using co-cultured human epidermal keratinocytes (HEK) and human dermal fibroblasts (HDF) in SM and ALI microenvironments.

## 2. Materials and Methods

This study was approved by the Universiti Kebangsaan Malaysia (UKM) Research Ethics Committee (Code No. FF-2015-087).

### 2.1. Extraction and Purification of Ovine Collagen Type I

Ovine tendon collagen type I (OTC-I) was extracted and purified as previously described by Fauzi and co-workers (2016) [21]. Crude tendon was cleaned of fascia and muscle tissues and freeze-dried for 48 h. The dried tendon was cut into small pieces and dissolved in 0.35 M acetic acid (VWR, Leicestershire, UK) at 4 °C for 24–48 h to extract the collagen protein. Sodium chloride (0.05 g/mL; Sigma, St. Louis, MO, USA) was added to the collagen solution and incubated at 4 °C for 24–48 h, followed by centrifugation at 10,000 rpm for 45 min. The collagen pellet was then dialysed for 72 h using dialysis tube (molecular weight cut off = 14 kDa) (Sigma, St. Louis, MO, USA) with alternate changes of disodium hydrogen phosphate solution (Na_2_HPO_4_; 0.2 M) and phosphate buffered saline (PBS; 1x) as the dialysis buffer every 12 h. Dialysed collagen was frozen at −80 °C for 6 h followed by freeze-drying for 24–48 h [21]. OTC-I then was re-dissolved in 0.35 M acetic acid overnight at 4–8 °C for a final concentration of 25 mg/mL.

### 2.2. Fabrication of OTC-I Sponge Scaffolds

The OTC-I solution was poured into the desired mould and frozen at −80 °C for 6 h, followed by a freeze-drying process (Ilshin, Gyeonggido, Korea) for 24–48 h. Commercialised atelocollagen (ATC) was used as a control.

### 2.3. Crosslinking of OTC-I Sponge Scaffolds

OTC-I sponge scaffolds were crosslinked with 0.1% (*w*/*v*) genipin (GNP; Wako, Osaka, Japan), which is a natural crosslinking agent and 1 M (*w*/*v)* 1-ethyl-3-(3-dimethylaminopropyl) carbodiimide (EDC: Gibco, Waltham, MA, USA), which is a synthetic crosslinking agent. The sponge scaffolds were dissolved in the GNP and EDC working solution at room temperature for 6 h, followed by several washing steps using PBS. Furthermore, both the crosslinked scaffolds were then frozen at −80 °C for 6 h prior to freeze-drying for 24–48 h before use for further analysis. The OTC-I sponge crosslinked with genipin (GNP) and carbodiimide (EDC) were denoted as OTC-I_GNP and OTC-I_EDC sponge, respectively. A non-crosslinked OTC-I sponge and atelocollagen (ATC) were used as the controls.

### 2.4. Characterisation of OTC-I Sponge Scaffolds

All experiment was repeated with three biologically independent samples for each of the fabricated non-crosslinked and crosslinked scaffolds.

#### 2.4.1. Gross Appearance

The gross photos of fabricated non-crosslinked and crosslinked OTC-I sponges were taken by a digital camera. The colour appearance for all fabricated sponges was visualised and recorded. Furthermore, the non-crosslinked and crosslinked OTC-I sponge scaffolds were stained using picrosirius red (PSR; Polysciences, Warrington, PA, USA) for at least 1 h and until it reached near-equilibrium staining. The non-crosslinked and crosslinked OTC-I sponges were soaked in solution A (phosphomolybdic acid hydrate) for 2 min, in solution B (2,4,6-trinitrophenol) for 60 min and subsequently in solution C (0.1% hydrochloric acid) for 2 min as mentioned in the manufacturer’s protocol. Most of the excessive water from the slides was physically removed by vigorous shaking.

#### 2.4.2. Swelling Ratio and Water Uptake Ability

The swelling ratio was performed as mentioned in a previous publication by Yang and co-workers (2018) with minor modifications [24]. Freeze-dried non-crosslinked and crosslinked OTC-I sponges were initially weighed to obtain their dry weight (S_d_). Then, the scaffolds were immersed in PBS (pH 7.2) and incubated at 37 °C for 1 h. The excessive liquid from the sponge scaffolds surface were gently removed using filter paper, followed by weighing to obtain the weight after swelling (S_s_). The water uptake ability was evaluated after incubation for 48 h at 37 °C to permit water adsorption therefore as mentioned in previous publication [25] the sponges were initially weighed to obtain their dry weight (W_d_) followed by weighing to obtain their weight after water uptake (W_s_). The swelling ratio and water uptake were calculated using the equations below:

$$Swelling\;Ratio\;\left(\%\right)=\frac{S_{s}-S_{d}}{S_{d}}\times 100$$

$$Water\;Uptake\;\left(\%\right)=\frac{W_{s}-W_{d}}{\;W_{d}}\times 100$$

#### 2.4.3. Morphological Evaluation

The surface topography and cross-section microstructure of non-crosslinked and crosslinked OTC-I sponges were evaluated via scanning electron microscopy (SEM; FEI, Hillsboro, OR, USA) as mentioned in a previous study [23]. The fabricated bioscaffolds were fixed with 4% glutaraldehyde and dehydrated in a series of ethanol solutions at 30%, 50%, 70% and 100% (*v*/*v*) for 10 min each. Finally, the OTC-I sponges were dried overnight using a critical point dryer (CPD 030; Bal-Tec, Los Angeles, CA, USA) followed by nanogold coating. The scaffolds were observed by SEM and the pore size was measured randomly using Phenom Pro X integrated software (Phenom, Eindhoven, The Netherland).

#### 2.4.4. Fourier Transform Infrared Spectrometry

The chemical characterisation of the OTC-I sponges was performed using Fourier transform infrared spectrometry (FT-IR), as described in Busra et al. (2019) [23]. The scaffolds were cut into 1 mm^3^ pieces and the spectral data were recorded by a PE Spectrum 100 FT-IR spectrometer (PE, Waltham, MA, USA) in a wavelength range of 700 to 4000 cm^−1^. The absorbance peaks were analysed to identify any chemical structural changes after the crosslinking process.

### 2.5. Human Skin Cell Isolation and Culture

Redundant skin tissue samples were processed as previously described by Busra and co-workers (2019) [23]. A skin sample 3 cm^2^ in size was minced into small pieces and digested by dual enzymatic approaches with 0.6% collagenase type I (Worthington, Lakewood, NJ, USA) for 5–6 h at 37 °C in a shaker incubator, followed by cell dissociation using 0.05% trypsin-EDTA (Gibco, Waltham, MA, USA) for 10 min. The cell suspension was then centrifuged. Co-cultured skin cells were seeded in three wells of a six-well polystyrene plate (tissue culture treated; Greiner, Frickenhausen, Germany) and maintained at 37 °C in a 5% CO_2_ incubator, with medium changes every 2–3 days. Both co-cultured epidermal keratinocytes (HEK) and dermal fibroblasts (HDF) were cultured in an equal amount of serum-free Epilife medium (Gibco, Waltham, MA, USA) and F12:DMEM (Ham’s F-12 nutrient: Dulbecco’s Modified Eagle Medium) (1:1, Gibco) medium which was supplemented with 10% foetal bovine serum (FBS) (Biowest, Riverside, MO, USA), 1% antibacterial-antimycotic (AA) (Gibco, Waltham, MA, USA), 1% Glutamax (Gibco, Waltham, MA, USA) and 2% HEPES (Gibco, Waltham, MA, USA). Once skin cells reached 70–80% confluence, differential trypsinisation was performed to dissociate HDF from culture plate using 0.05% Trypsin-EDTA. The HDF were seeded in 75 cm^2^ culture flasks with F12:DMEM medium containing 10% FBS while HEK were cultured in a six-well plate with Epilife (Gibco, Waltham, MA, USA) medium.

### 2.6. Cell-Bioscaffold Interaction

#### 2.6.1. Cellular Toxicity

The live/dead cell viability assay (Life Technologies, Waltham, MA, USA) was used to analyse the cytotoxic effect of the non-crosslinked and crosslinked OTC-I sponges according to the manufacturer’s protocol. On the culture plate, co-culture HEK and HDF at a density of 1 × 10^4^ cells/cm^3^ were seeded on the surface of all of the fabricated bioscaffolds and incubated at 37 °C, with 5% CO_2_ for 24 h. The fabricated bioscaffolds seeded with co-culture HEK and HDF then were incubated with calcein AM (2 mM) and EthD-1 (4 mM) in PBS for 30 min as recommended by the manufacturer’s protocol. Subsequently, the stained cells were observed using a Nikon A1R-A1 confocal laser scanning microscope (CLSM; Nikon, Tokyo, Japan). The live and dead cells were stained green (calcein) and red (EthD-1), respectively.

#### 2.6.2. Cell Attachment

For cell attachment analysis, co-culture HEK and HDF at passage 2 were seeded on the non-crosslinked and crosslinked OTC-I sponges at a density of 1 × 10^4^ cells/cm^3^. Unattached cells in the culture suspension were quantified at 2 h after seeding using the trypan blue exclusion test. Subsequently, the percentage of cell attachment on the non-crosslinked and crosslinked OTC-I sponges was evaluated using the following equation:

$$\mathrm{Cell}\;\mathrm{Attachement}\;\frac{\mathrm{Seeded}\;\mathrm{cells}-\mathrm{Unattached}\;\mathrm{cells}\;}{\mathrm{Seeded}\;\mathrm{cells}}\times 100$$

#### 2.6.3. Cell Proliferation

A 1 × 10^4^ co-culture of HEK and HDF were seeded on the non-crosslinked and crosslinked OTC-I sponges in a 48-well culture plate for different time points (day 1, 3 and 5). The quantification of cell proliferation was performed by using the Vybrant^®^ MTT cell proliferation assay kit (Invitrogen, Waltham, MA, USA) and, hence, the manufacturer’s instructions were followed. At day 1, 3 and 5 the seeded sponges were transferred to a new 48-well culture plate to avoid the possibility of false positive results from the seeded cells that might have attached on the surface of the well. The spent medium was replaced with 100 µL of fresh co-culture medium containing Epilife-F12:DMEM at a 1:1 ratio. Then, 10 µL of MTT reagent was added into each well and incubated at 37 °C for 4 h. Next, 100 µL of 10% (*w*/*v*) SDS in 0.01 M HCl solution was added into each well, followed by 4 h of incubation. Finally, 100 µL of the solution was transferred into a 96-well plate and the absorbance was read at 570 nm using a spectrophotometer (Bio-Tek, Power Wave XS, Winooski, VT, USA).

### 2.7. In Vitro Reconstructed Skin Model

#### 2.7.1. Submerged Microenvironment

The non-crosslinked and crosslinked OTC-I sponge scaffolds were inserted into a 24-well culture plate. The co-culture HEK and HDF were then seeded onto the fabricated sponges with a surface area of 0.33 cm^2^ and a density of 5 × 10^5^ cells/cm^2^. For the submerged microenvironment, the co-cultured HEK and HDF were maintained as follows: co-culture medium containing Epilife (Gibco, Waltham, MA, USA) and F12:DMEM in a 1:1 ratio was added in each well to a volume such that the medium level reached slightly higher than the sponge’s height. The scaffolds seeded with co-cultured HEK and HDF were maintained in an incubator with 5% CO_2_, at 37 °C and the medium was changed on a daily basis for 30 days of the culture period.

#### 2.7.2. Air-Liquid Interface (ALI) Microenvironment

The seeding of co-cultured HEK and HDF was done similar to the submerged conditions and the seeded scaffolds were maintained in a submerged microenvironment for two days. Afterward, to initiate the air-liquid interface (ALI) microenvironment, the media was completely aspirated from the 24-well culture plate and from inside the culture insert. Then, fresh co-culture medium containing Epilife (Gibco, Waltham, MA, USA) and F12:DMEM in a 1:1 ratio was added to the lower compartment (culture plate), allowing the co-culture HEK and HDF to be exposed to the air from the surface and for the media to be perfused only from the lower side. To allow the co-cultured cells on the surface to remain exposed to the air, subsequent media replacement for the next 30 days was performed in the lower compartment only.

#### 2.7.3. Morphology Assessment

The non-crosslinked and crosslinked OTC-I sponges were seeded with co-cultured HEK and HDF and remained in culture for 30 days. Next, the cells were fixed with 3% glutaraldehyde and a serial chemical process and observed via SEM for the microscopic evaluation of cell morphology. The morphological evaluation was done separately for submerged conditions and the ALI microenvironment.

### 2.8. Statistical Analysis

The statistical evaluation was performed using GraphPad Prism 5.0 (GraphPad Software, San Diego, CA, USA). The data are expressed as the mean ± standard deviation (SD). The comparison between multiple groups was analysed by ANOVA and any statistically significant difference was deemed when *p* < 0.05.

## 3. Results

### 3.1. Physical Characterisation of OTC-I Bioscaffolds

#### 3.1.1. Gross Appearance

**Figure 1:**
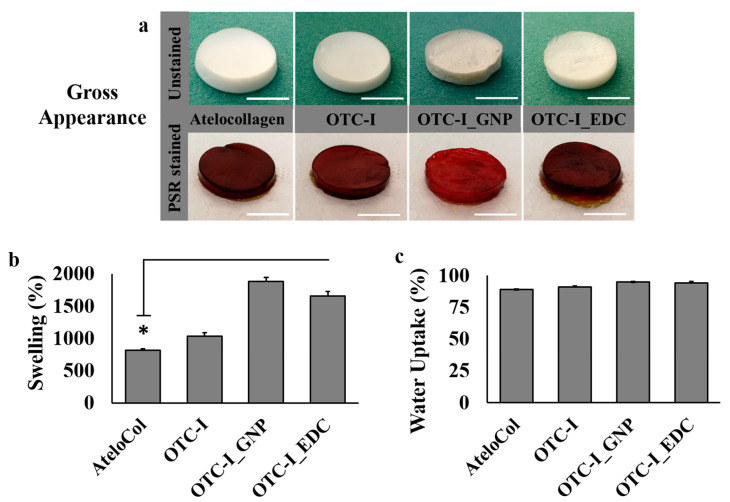
Gross appearance (**a**), and physical properties including the swelling (**b**) and water uptake (**c**) of the fabricated ovine collagen bioscaffold (white bar = 10 mm). * represents statistically significant difference (*p* < 0.05).

The gross appearance of atelocollagen (ATE), non-crosslinked and crosslinked (OTC-I) sponges is illustrated in Figure 1a. In the unstained condition, the ATE, OTC-I and OTC-I_EDC sponges appeared white, as seen in Figure 1a. However, the OTC-I_GNP presented as a light brown sponge. The ATE, OTC-I and OTC-I_EDC sponged were visualised as dark red in colour after staining with picrosirius red (PSR). However, the OTC-I_GNP construct appeared light red, as seen in Figure 1a.

#### 3.1.2. Swelling Ratio and Water Uptake Ability

The swelling ratio and water uptake analysis were determined as shown in Figure 1b,c, respectively. The OTC-I_GNP sponge scaffold demonstrated the best swelling property (1886% ± 56%) compared to the other experimental groups. Meanwhile, the ATE sponge had the poorest (813% ± 66%) swelling property, followed by non-crosslinked OTC-I (1033% ± 30%) and OTC-I_EDC (1658% ± 62%). The water uptake ability of all fabricated sponges was higher than 75%, as illustrated in Figure 1c. There was no significant difference between all treatment groups compared to the ATE sponge, even though OTC-I_GNP demonstrated a slightly high percentage of water uptake.

#### 3.1.3. Morphological Evaluation

**Figure 2:**
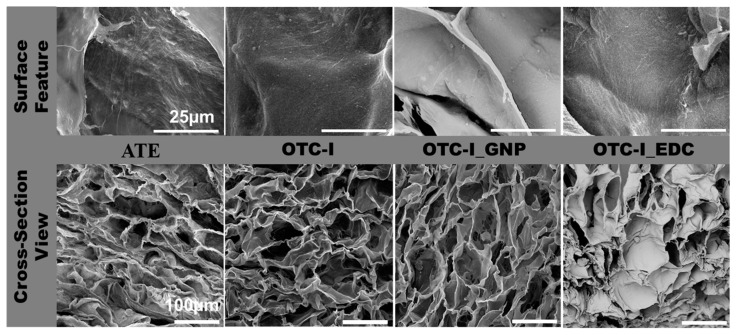
The surface topography and microporous structure of fabricated ovine collagen bioscaffold via scanning electron microscopy. All fabricated collagen bioscaffolds demonstrated suitable surface roughness potentially for cell attachment (collagen fibrils) and high porosity.

The photomicrographs of surface topography and cross-sections of the fabricated sponges were examined via scanning electron microscopy (SEM), as shown in Figure 2. Tiny fibre-like microstructures on the surface of all fabricated sponges (Figure 2) were clearly observed. However, those fibre-like structures were notably more prominent in terms of visibility on the ATE and non-crosslinked OTC-I compared to OTC-I_GNP and OTC-I_EDC. SEM was further used to evaluate the cross-sectional micromorphology of all fabricated sponges (Figure 2). The density of pores was similar between ATE and OTC-I_EDC. Additionally, the density of pores was similar between OTC-I and OTC-I_GNP. Meanwhile, the depth of pore structures in non-crosslinked OTC-I was similar to other sponges. All fabricated sponges presented a heterogenous pore size with pore size range from 100 to 200 μm. Moreover, porosity evaluation revealed that OTC-I_EDC has fewer interconnected pores than non-crosslinked OTC-I and OTC-I_GNP (both data were published elsewhere [23]). Visual inspection revealed no discernible differences in pore structure between groups. All groups displayed a heterogenous porous structure with no apparent alignment in any direction.

### 3.2. Chemical Characterisation of OTC-I Bioscaffolds

#### Fourier Transformation Infrared (FT-IR)

**Figure 3:**
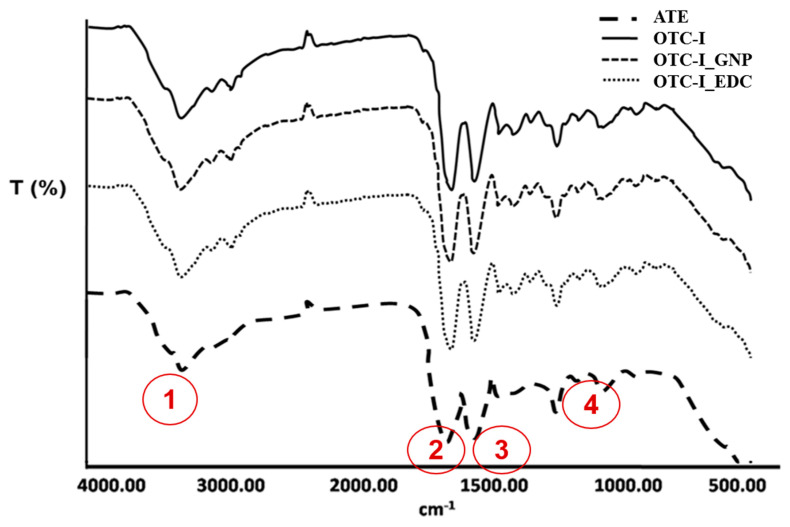
Chemical characterization of collagen bioscaffold via Fourier transformation infrared (FTIR). The fabricated collagens showed the amide A (1), I (2), II (3) and III (4) for collagen characterization.

Chemical characterisation of the non-crosslinked and crosslinked OTC-I sponge scaffolds was performed using FT-IR spectrometry. The FT-IR spectra of fabricated sponges are illustrated in Figure 3. The spectra demonstrated four visible absorbance peaks at different frequencies. The FT-IR spectrum of OTC-I demonstrated absorbance peaks for amide A due to N–H stretching (3300 cm^−1^), amide I due to C–O stretching (1634 cm^−1^), amide II due to N–H deformation (1547 cm^−1^) and amide III due to N–H deformation (1235 cm^−1^), which correspond to the characteristics of collagen type I. Obviously, there was no major shift in the FT-IR spectra of OTC-I_GNP and OTC-I_EDC regarding the absorbance peaks of amide A, I, II and III, as presented in Figure 3. Moreover, the detected peaks in non-crosslinked and crosslinked OTC-I were consistent with those of the ATC control.

### 3.3. Cellular Biocompatibility

#### 3.3.1. Cell Cytotoxicity

**Figure 4:**
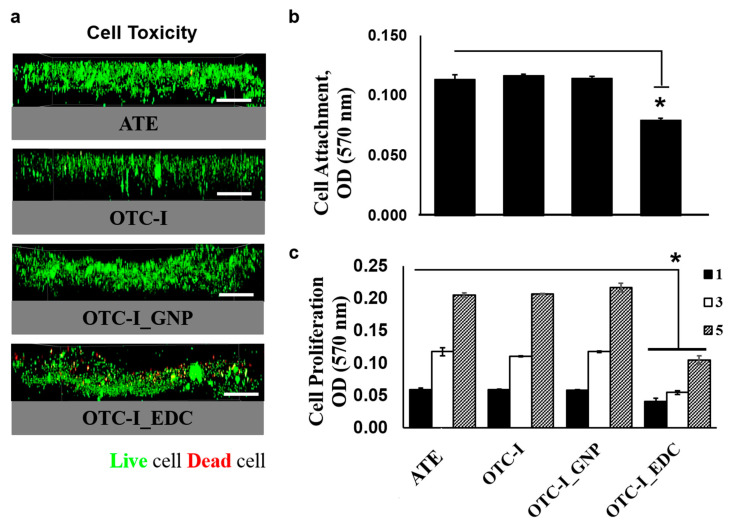
Immunochemical (**a**) and the HDF attachment (**b**) and growth pattern (**c**) of skin cells seeded on fabricated collagen bioscaffold at day 1, 3 and 5 (bar = 100 μm). The skin cells viability well-distributed on each collagen bioscaffold except the group crosslinked with carbodiimide (EDC). The skin cells growth gradually increased following time for all treatment groups and significantly lower in the group crosslinked with carbodiimide (EDC). * represents statistically significant difference (*p* < 0.05).

Co-cultured human epidermal keratinocytes (HEK) and human dermal fibroblasts (HDF) were seeded on the non-crosslinked and crosslinked OTC-I sponge scaffolds to evaluate their cytotoxicity (Figure 4a); ATC was used as a control. Figure 4a demonstrates that, with the exception of OTC-I_EDC, all of the fabricated sponges showed no cytotoxic effect towards HEK and HDF. In the fluorescent images of non-crosslinked OTC-I, OTC-I_GNP and ATC sponges, the seeded HEK and HDF were visualised in green only (representing viable cells). The results revealed that the fabricated scaffolds ad no cytotoxic effect on co-cultured HEK and HDF. In contrast, in fluorescent image of HEK and HDF seeded on OTC-I_EDC were green as well as red (representing dead cells), which suggests that the OTC-I_EDC scaffold demonstrated some level of toxicity towards co-cultured HEK and HDF.

#### 3.3.2. Cell Attachment

The attachment of co-cultured HEK and HDF 24 h post-seeding on the non-crosslinked OTC-I, OTC-I_GNP, OTC-I_EDC and ATC sponges are shown in Figure 4b. The results revealed no significant differences between non-crosslinked OTC-I and OTC-I_GNP compared to the ATC sponge. In contrast, the OTC-I_EDC sponge demonstrated significantly less cell attachment than the other fabricated sponges. The results indicate that the non-crosslinked OTC-I, OTC-I_GNP and ATC sponges could provide a favourable microenvironment for cell attachment with non-cytotoxic effects.

#### 3.3.3. Cell Proliferation

The proliferation of co-cultured HEK and HDF on the non-crosslinked OTC-I, OTC-I_GNP and OTC-I_EDC sponges was evaluated at different time points: day 1, 3 and 5 (Figure 4c). The ATC sponge was used as a control. Each sponge demonstrated a gradual increase in cell proliferation from day 1 to day 5. However, no significant differences were observed in the proliferation of seeded HEK and HDF on non-crosslinked OTC-I, OTC-I_GNP and ATC sponges at different time points of evaluation. However, the OTC-I_EDC sponge demonstrated significantly less cell proliferation at different time points as compared to the other groups. This result suggests that co-cultured HEK and HDF proliferated better on the non-crosslinked OTC-I, OTC-I_GNP and ATC sponges as compared to the OCT-I_EDC sponge. The cell proliferation assessment of seeded HEK and HDF on the OTC-I_EDC sponge are consistent with the cytotoxic and cell attachment properties of the OTC-I_EDC sponge, which further indicates the unsuitability of the OCT-I_EDC sponge for HEK and HDF co-culture.

### 3.4. Micromorphology under Submerged and Air-Liquid Interface Microenvironments

**Figure 5:**
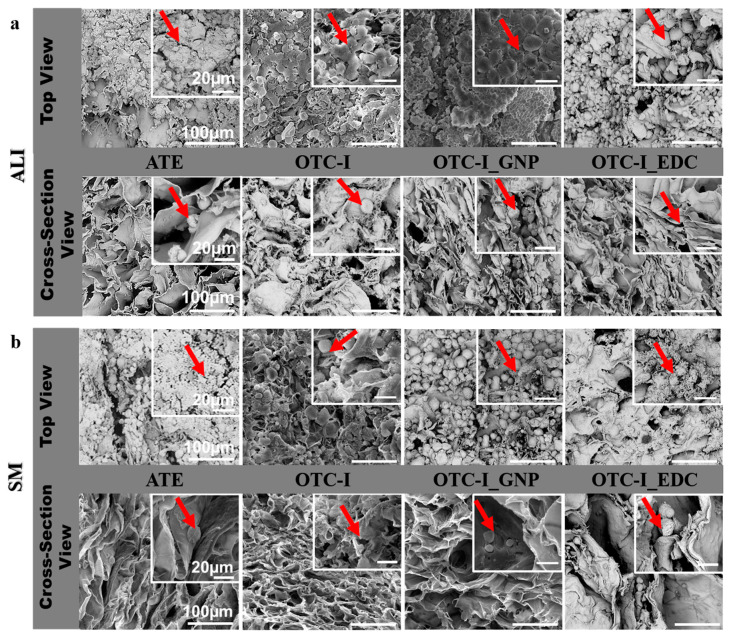
Morphological features of skin cells under (**a**) air-liquid interface (ALI) and (**b**) submerge (SM) condition via scanning electron microscopy. Top view referring the keratinocytes colonization and migrated fibroblasts in the cross-section view. Different morphology of fibroblasts has been visualized that in round shape with matrices production in surrounding area.

The evaluation of co-cultured HEK and HDF on the fabricated OTC-I sponges is illustrated in Figure 5 using scanning electron microscopy (SEM). Briefly, all sponges were seeded with co-culture HEK and HDF on the top level, followed by prolonged culture under submerged (SM) and air-liquid interface (ALI) conditions for 30 days. Generally, all sponges promoted cell adhesion and migration for both types of skin cells. Nonetheless, both HEK and HDF seeded on the OTC-I_EDC sponge in both SM and ALI microenvironments presented an apoptotic-like morphology with less matrix produced in the surrounding area. In addition, in the top layer view of OTC-I_EDC, no mature HEK were observed; no polygonal shapes and no round morphology were observed. Additionally, in OTC-I_EDC sponge, no cell-cell integration and less cell growth were observed as compared to the other sponges (especially under ALI conditions) (Figure 5a). In the SM microenvironment and on top layer of non-crosslinked OTC-I and OTC-I_GNP sponges, HEK demonstrated less cell-cell integration, a moderately round morphology and less matrix production than that on the ATC sponge (Figure 5b). In the ALI microenvironment, there was a major difference in HEK morphology on the non-crosslinked OTC-I, OTC-I_GNP and ATC sponges. Interestingly, all HEK in ALI visualised good cell-cell interactions, a polygonal morphology and matrix production in the surrounding area. The OTC-I_GNP sponge was found to be covered by HEK on most of the surface area (Figure 5b). The cross-sectional view displayed HDF migration in all groups with matrix production in both SM and ALI conditions.

## 4. Discussion

The goal of this study was to fabricate a three-dimensional (3D) in vitro skin model or reconstructed human skin (RHS) comprising of ovine tendon collagen type I (OTC-I) as a bioscaffold seeded with co-culture HEK and HDF. Development of an RHS should take into consideration several factors, including a suitable provisional bio-template, cell type, microenvironment and related culture medium. Direct contact of HEK with HDF (co-culture) is critical for the proliferation, migration and stratification of the epidermis [26]; therefore, the idea for this study was based on exploring the potential of a one-step seeding approach in which the co-cultured HEK and HDF were seeded onto the fabricated bioscaffold in a submerged (SM) or air-liquid interface (ALI) microenvironment to form a 3D in vitro skin model. Since it is no longer allowed to test a new product for the market using an animal model [27], using this novel 3D in vitro skin model could potentially replace animal models.

Previous studies have shown that treatment of collagen with genipin induces colour changes from yellowish to the dark bluish range dependent on treatment duration. The colour changes of collagen indicated successful cross-linking of genipin with primary amines [28], and can explain the colour difference of the OTC-I_GNP construct compared to the others. It has also been shown previously that genipin treatment strengthens collagen biomechanics through crosslinking intramolecular and intermolecular covalent bonds in collagen fibres [28], which improves stiffness [29], and the stability of collagen fibres and maintains fibre morphology when they come into contact with an aqueous environment [30]. Being able to maintain the mechanical integrity of a construct is a beneficial feature since it suggests that the construct can serve as a viable in vitro wound healing model for at least one week. As it was shown in this study, the OTC-I_GNP construct demonstrated the highest swelling capacity as compared to other constructs; and this phenomenon is due to the effect of genipin cross-linking with OTC-I. It was proven in our previous study [23] that genipin cross-linking with OTC-I increases the stiffness of the construct, but the flexibility and swelling capacity of OTC-I_EDC were lower compared to OTC-I_GNP.

The swelling ratio is an important parameter because it indicates how the sponge scaffold will respond when it comes into contact with blood or other body fluids; this can also affect the cell differentiation process [31]. The swelling ratio shows the ability of the sponge to absorb liquid. Sponge scaffolds with a high swelling ratio are useful for highly exudative wounds, as the sponge will absorb excess wound exudates. Water uptake ability is also an essential scaffold property because excess water absorption may expand and deform the scaffold. Water uptake ability is also closely related to porosity. A reduced water uptake ability may be related to decreased porosity [32]. In tissue engineering, the porosity of the scaffold is an important factor for cell migration and nutrient supplementation [32]. The three-dimensional microstructure of the ATE, non-crosslinked and crosslinked OTC-I sponge scaffolds were evaluated using SEM, which indicated that crosslinking of collagen with GNP and EDC affected the porosity of the collagen sponge. These results are consistent with previous studies conducted by Amri et al. (2014) [33], and Fauzi et al. (2019) [23].

As described in our previous study, the FT-IR peak at 1635 cm^−1^ indicated that OTC-I had a β-sheet and triple helix structure. Moreover, the peak intensity at 1546 cm^−1^ to 1235 cm^−1^ indicates the triple helical content in collagen. The results were consistent with previous studies conducted by Riaz et al. (2018) [34], and Fauzi et al. (2016) [21], that performed an FT-IR analysis of different collagen sources. The characteristics of collagen should be preserved to ensure that the microenvironment can support the attachment, proliferation, migration and differentiation of both skin cell types.

Cell toxicity in evaluating the suitability of construct in supporting the attachment and growth of the seeded cells demonstrated that the non-crosslinked OTC-I and OCT-I_GNP were equally favourable for cell attachment and they did not interfere with cell growth. Cell toxicity associated with genipin had been found to be dose-dependent [35], so these favourable results for a genipin concentration of <1.0 mM and treatment time frame of <24 h were expected and are consistent with previously reported data [36].

The cytotoxicity and unfavourable properties of OTC-I_EDC in supporting cell attachment and proliferation might be primarily due to the interaction of EDC with collagen binding sites. Previous studies have shown that EDC-crosslinked collagen scaffolds consume carboxylate-containing acid side chains, which are essential for recognition by the cell surface. Thus, it adversely affects the attachment of cells to the scaffold through collagen-binding integrins [37,38]. A low concentration of EDC for crosslinking allows for retaining the native-like integration of cells to collagen-binding integrins, as shown in a previous study [39]. However, there is no consensus on this matter in the literature, and there are plenty of reports demonstrating the cytocompatibility of EDC cross-linked collagen-based scaffolds for fibroblasts and keratinocytes [40,41]. Therefore, to have a comprehensive view on the effect of EDC, parameters including the serum content (cell adhesion factors) of culture media and culture duration should be taken into consideration [42]. In long-term culture, the initial response of cells to the biomaterial might be altered since cells often undergo remodelling over time and secrete their own extracellular matrix [43], which could change their behaviour towards the biomaterial and facilitate their attachment to the material.

To date, there are no data available on the direct measurement of changes in cell polarisation in response to their surrounding 3D-environment and the mechanical forces generated by neighbouring cells. However, Giavazzi and co-workers in 2018 reported that a change in the surrounding environment induced direct effects on the collective migration of epithelial cells. This cell flocking caused the rearrangement of cells and induced the alignment of cells perpendicularly to the migration direction [44]. A similar concept had been demonstrated by Bernstam et al. in 1986, in that culturing keratinocytes an air-liquid interface resulted in the formation of a thick and multilayered sheet of cells which resembles native skin with features including spatial organisation, keratohyaline-like granules, desmosomes, interdesmosomal dense lines and more than 20 non-nucleated cell layers which resembles the stratum corneum in vivo [45]. Therefore, the air-liquid interface (ALI) microenvironment provides a sense of direction for keratinocytes and mimics the native microenvironment of skin, which induced the continuous and collective migration of HEK towards the air phase. Therefore, it was expected that the newly proliferated keratinocytes migrate upwards and undergo a programmed differentiation into a “brick and mortar”-like structure which ensures the barrier function of the epidermis [23]. This ALI approach provided a more realistic 3D skin model for future investigations; the submerged constructs were promising for cell attachment and proliferation, but not for differentiation.

The observation of an effect of the submerged and ALI microenvironments with the micromorphology of seeded HEK and HDF were consistent with the cytotoxicity results. In both microenvironment conditions, the OTC-I_EDC sponge did not favour the seeded cells and imposed toxicity on skin cells, followed by apoptosis. We found a synergistic enhancement of cell growth in HDF and HEK co-culture; however, this observation was in contrast with previous findings by one of the co-authors in which the absence of cell growth in co-cultured constructs was reported [46]. This work serves as a foundation for understanding some of the necessary factors needed to develop a stable and functional model of full-thickness skin to cater to future applications, such as drug and herbal remedy testing. The preliminary results obtained in this study would be improved in the near future to enhance the clarity of current findings including the histology and immunohistochemistry outputs.

## 5. Conclusions

In conclusion, these preliminary results provide valuable information on a three-dimensional (3D) in vitro skin model using ATE, non-crosslinked and crosslinked OTC-I sponge scaffolds seeded with HEK and HDF in submerged (SM) and air-liquid interface (ALI) microenvironments. Even though OTC-1_GNP was found to be the optimal construct for supporting co-cultured HEK and HDF in an ALI microenvironment and could be a potential candidate for future 3D skin reconstruction modelling. Moreover, to improve the current findings, the authors suggest further histological and immunohistochemical analysis, to confirm multi-layer formation and stratification of keratinocytes and the detection of keratinocytes terminally-differentiated markers, respectively. In future models of 3D skin, to further stimulate multi-layer keratinocyte formation, culturing HEK in higher concentrations of Ca^+^ ions and lower incubation temperatures can be investigated.
